# Exergy Analysis Using a Theoretical Formulation of a Geothermal Power Plant in Cerro Prieto, México

**DOI:** 10.3390/e23091137

**Published:** 2021-08-31

**Authors:** Dario Colorado-Garrido, Gerardo Alcalá-Perea, Francisco Alejandro Alaffita-Hernández, Beatris Adriana Escobedo-Trujillo

**Affiliations:** 1 Centro de Investigación en Recursos Energéticos y Sustentables, Universidad Veracruzana, Av. Universidad km 7.5, Col. Santa Isabel, Coatzacoalcos C.P. 96535, Veracruz, Mexico; galcala@uv.mx (G.A.-P.); falaffita@uv.mx (F.A.A.-H.); 2 Facultad de Ingeniería, Universidad Veracruzana, Av. Universidad km 7.5, Col. Santa Isabel, Coatzacoalcos C.P. 96535, Veracruz, Mexico; bescobedo@uv.mx

**Keywords:** geothermal energy, net power output, exergy destruction, thermodynamic assessment

## Abstract

The purpose of this research is the calculation of the exergy destruction of the single-flash and double-flash cycles of a geothermal power plant located on the ladder of the 233 m Cerro Prieto volcano, on the alluvial plain of the Mexicali Valley, Mexico. The methodology developed in this research presents thermodynamic models for energy and exergy flows, which allows determining the contribution of each component to the total exergy destruction of the system. For the case-base, the results indicate that for the single-flash configuration the efficiency of the first and second law of thermodynamics are 0.1888 and 0.3072, as well as the highest contribution to the total exergy destruction is provided by the condenser. For the double-flash configuration, the efficiency of the first and second law of thermodynamics are 0.3643 and 0.4983. The highest contribution to the total exergy destruction is provided by the condenser and followed by the low-pressure turbine.

## 1. Introduction

Although Mexico has a National Interconnected Electrical System (NIES), there are three additional isolated electrical systems in the country that supply 7.1% of the energy: the Electrical System of Baja California Sur, the Electrical System Mulegé and the Electrical System of Baja California [1]. In fact, the electrical system of Baja California is connected to the Western Electricity Coordinating Council in the United State via two permanent interconnections (Tijuana-Miguel and La Rosita Imperial Valley) that allow the import and export of energy.

The supply of electricity in the Baja California region is made up of several types of power plants: combined cycle, turbo-gas, natural-gas diesel internal combustion and geothermal. In 2019, 14 utility-scale power plants were operational in Baja California with a combined installed capacity of 4049 MW, with 1102 MW destined for export to California, that left Baja California with an installed capacity of 2947 MW [2] out of which the geothermal power plants of Cerro Prieto made up 13% of total effective capacity in 2018.

**Figure 1:**
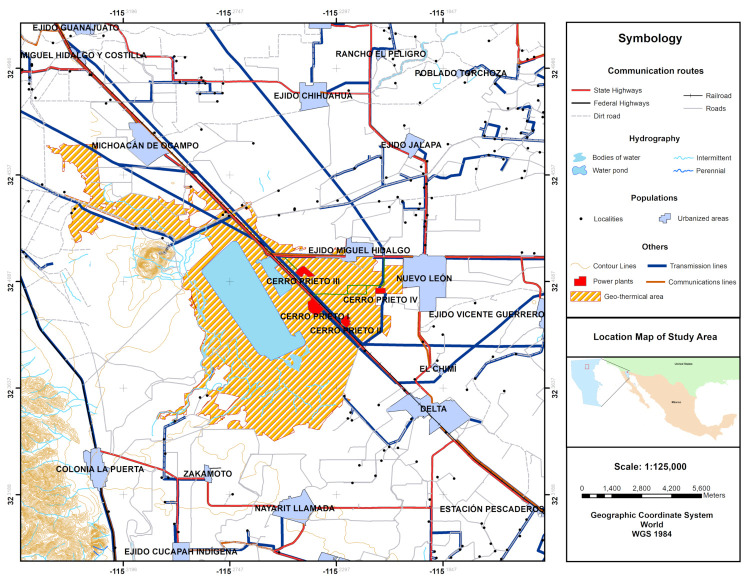
Cerro Prieto Geothermal Power Plant location.

The geothermal field of Cerro Prieto, is located on the mountainside of the 223 m Cerro Prieto volcano, on the alluvial plain of the Mexicali Valley, between the meridians 115${}^{\circ }$12*’* and 115${}^{\circ }$18*’* W and the parallels 32.22${}^{\circ }$ and 32${}^{\circ }$26*’* N, 32 km from the US-Mexico western border (see Figure 1). It is the largest of the four Mexican fields in commercial exploitation, and the second largest worldwide, covering an area of 18 km${}^{2}$, although the reservoir reserves in the subsoil extend to cover other 50 km${}^{2}$ [3]. Currently, Cerro Prieto is operating at a little less than 60% of its installed capacity and less than 50% of its original capacity [2], with 570 MW, divided into 4 operating areas, CP I (30 MW), CP II (220 MW), CP III (220 MW), CPIV (100 MW) [1]. Nevertheless there are areas which the National electricity commission (CFE, in Spanish) has explored in order to locate medium-term geothermal resources that could increase reserves, these are located in the western part of the Geothermal Field of Cerro Prieto in the areas of Tulecheck, Laguna Salada, Lado east of the Sierra Cucap [4].

Given its importance, several studies have been devoted to study the Cerro Prieto Geothermal Plant (CPGP), focusing on different aspects, such as the geothermal and volcanic characteristic, thermodynamic model of plant and pipeline network, heat flux and hydraulic aspects, quality water and influence of pollution of Cerro Prieto Geothermal Plant, all of the above supported by geographical maps and in, some cases, geographical information systems (GIS), i.e., Prol-Ledesma and Morán-Zenteno [5] developed the characterization of geothermal regions in Mexico following several criteria: the geothermal displays, the patterns observed in the heat of data, the geological and tectonic features and surface display. The authors present the map of geothermal provinces in Mexico and classify in the CV3-CP province of the Cerro Prieto system assuming characteristics as convection-dominated extensional domain and continental rifting process related to the Gulf of California opening. According to the assessment, the average heat flow is close to 200 $\frac{mW}{m^{2}}$ with a geothermal gradient of 100 $\frac{{}^{\circ }C}{km}$. Heat flow, hydrothermal manifestations, volcanic activity and tectonic features were plotted using Geographic Information System by [5] for CV3-CP regions, it includes the Cerro Prieto and Las Tres Virgenes characteristics.

Aguilar et al. [6] measured the ambient levels of $H_{2}S$, $SO_{2}$ in the region near CPGP, in the conclusions the authors suggest that background $SO_{2}$ levels impacted by the emissions for the geothermal plant, the mechanism found is the oxidation of $H_{2}S$ to $SO_{2}$. García-Gutierrez et al. [7] presented a hydraulic and numerical simulation using frictional losses and the Darcy-Weisbach friction factor of the Cerro Prieto geothermal field steam pipeline network, it has an approximate length of 125 km, the pipes are thermally insulated and the pipeline network was plotted on a map in the entire geothermal field. Armienta et al. [8] planned an experimental campaign in 2010 and 2011 and they analyzed 87 water samples from shallow and deep wells in the agriculture zones, irrigation canals, geothermal production wells, the evaporation pond and piezometers, all of them related to Cerro Prieto Geothermal power plant. The instruments were calibrated and advanced analytical techniques were followed to determine major ions, As, Cd, Pb, Cr, Cu, $SiO_{2}$, Li and others in order to find its influence on water quality, the authors mentioned that the drinking water is adequate for human consumption except for the presence of Fe and Pb in the hot season. Geographic information systems were applied by [8] to develop maps and contours to delimit the regions of influence of potential contaminants. Geological aspects [9], volcanic activity and geothermal research [10] are presented by several authors in the literature. As for the power production plant, the thermodynamic simulation using Hysys software commercial package was carried out by [11]. The model considers the simulation of the single and double-flash cycles in order to obtain the net power output of 36.6 MW and 102.1 MW respectively. The well temperature, separator pressure and condenser pressure are underlined as independent operation variables to predict net power outputs and the overall plant efficiency assuming a complete multivariate linear regression analysis.

The exergy analysis has been considered as a methodology that allows obtaining information on the performance and quality of the energy that is transformed into geothermal power cycles, this methodology estimates the degree of exergy destruction of each of pieces of equipment of a power plant. In the geothermal field, several authors have presented exergy results, the following is a bibliographic review in chronological order with studies that refer to exergy analysis developed in geothermal power plants. An exergy analysis by [12] was performed based on Still water binary geothermal power plant located in Northern Nevada, USA. The system analyzed by the author provides the net power output of 2159 kW and according the exergy analysis, the turbines and condenser represents 51.7% of the total exergy destruction of the overall system. A single-flash and a double-flash geothermal power plants are included in the exergy methodology by [13], the high and low pressure separator intervals of pressure were from 500 to 800 kPa and from 40 to 120 kPa respectively. According to [13], for the single stage-flash system, the greatest exergy destruction was found in the reinjection step and for the double stage-flash system, the turbines carries 17.27% of the total exergy destruction of the system. Jalilinasrabadya et al. [14] presented a single and double-flash geothermal power plants analysis using the energy and exergy concepts for the engineering conditions of Sabalan, Iran. For the authors, the total exergy destruction for single and double effect configurations were estimated in 111,781 kW for 31 MW of net power output and 114,832 kW for 49.7 MW of net power output, respectively. The contribution of each component to the total exergy destruction was computed and illustrated in Grassman plot. Cardemil et al. [15] presented a thermodynamics analysis using the first and second laws for the performance of single and double-stage geothermal power plants coupled by a parabolic through solar concentrating collector field, the influence of second law efficiency was discussed in every configuration allowed to identify the best engineering solution in the functioning of reservoir conditions, in all previous economic analyses were carried out based on the cost of electricity in northern Chile. Rudiyanto et al. [16] presented the exergy assessment of the Kamojang geothermal power plant in Indonesia, the exergy destruction of each component of the entire cycle was tabulated and discussed, the main condenser was designated as the most inefficient components around of 180 MW of exergy destruction, additionally the authors presented the influence of the environmental temperature on the exergy destruction in the turbine. Bina et al. [17] compared the single and double-flash systems for real data of geothermal conditions of Sabalan field following the energy, exergy and economic aspects. The exergy destruction at different stages of the single and double flash power plant were tabulated; it is interesting to note that the turbine for the single and double stage is the piece of equipment with the most significant contribution to the total exergy destruction. A co-generation system considering single-flash geothermal power plant, a photovoltaic thermal (PVT) and the double effect absorption chiller is optimized by [18] assumed energy and exergoeconomic considerations, the condenser in the geothermal system is identified as the component with the greatest exergy destruction in the entire system. A single stage geothermal power plant has been involved in co-generation concept, power along with hydrogen, both were outputs of the system simulated by [19]. From all of the above, it is interesting to note that the process or pieces of equipment that contributes in a greater amount to total exergy destruction changes according to the operating conditions and characteristics of the geothermal field under study.

We know that the main indicator in the geothermal plant is the net power output; in many cases it determines cycle operation decisions or the handling of the working fluid coming from the geothermal field. This research proposes to carry out a study based on the exergy analysis to provide information that allows engineers and designers to make decisions about the operating parameters of the geothermal plants to reduce the exergy destruction and define actions to increase thermal efficiency. The objective of this research is to provide a study of the quality of energy through first and second laws of thermodynamics of a single and double-flash cycles in Cerro Prieto geothermal power plants situated in Baja California Norte, Mexico. The theoretical tools that this research uses are: mass and energy conservation, exergy balances, pressure drop in steam transport, an appropriate calculation of thermo-physical properties of water, which was programmed in a free license software Octave language. The above information allows us to calculate the total exergy destruction of the cycles, as well as, identify the component that contributes the greatest exergy destruction to each cycle, which will allow the definition of technological strategies or operating alternatives. According to Hossein-Zolfagharnasab et al. [20], the code generated by GNU Octave allows us to program the energy conservation and exergy balance of a similar energy system with precision and in a friendly way.

The differences and novelties between studies done by [11,13,14,17,21] and the present study are:

1. The present manuscript considers the works done by [11,13,14,21]; however, the difference in this work is the calculation of the exergy destruction of the Cerro Prieto geothermal power plant assuming real operation variables immersed in the Mexican context. The above allows us to point out the piece of equipment that has the greatest contribution to exergy destruction. The main capacity of the exergy analysis is to quantify the exergy destruction of the processes involved in the energy resource conversion processes with reference to the geothermal power plant in Cerro Prieto, Mexico.
2. The authors of [14,17] provides thermodynamic formulation and report the influence of thermodynamic variables for single and double flash geothermal power plant from the exergy point of view. Additionally, this study estimates the flow of external cooling water that is consumed in the condenser and the influence of some operating conditions on it.

## 2. Single and Double Flash Cycles Description

**Figure 2:**
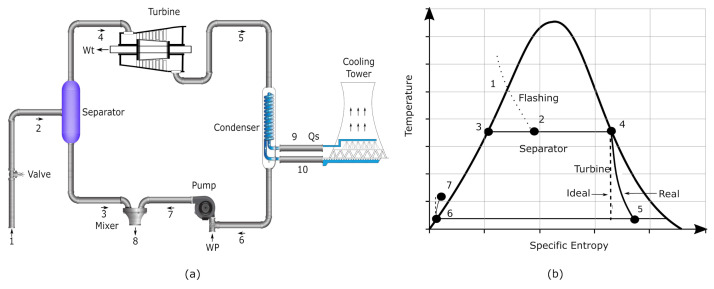
Schematic diagram of a single-flash configuration.

Figure 2a illustrates the schematic diagram of a single flash geothermal power plant and Figure 2b shows the entropy vs temperature diagram which shows the main processes involved in the cycle, the cycle is assembled with six components: a valve, a separator, a turbine, a condenser, a pump and a mixer. Impurity free Geothermal water is used as working fluid in the entire system. The geothermal working fluid goes through the valve as two phase flow with a quality close to saturated liquid, at the pressure of the separator the two-phase flow is separated in liquid and vapor steam, the flashing pressure in this point is mixed and thus determining the production of electrical energy. The saturated vapor steam goes through the turbine and it is expanded to produce useful work. The geofluid pressure is diminished and changes to liquid phase in the condenser. The flow is then pumped and goes to the mixer. A partial separation of the geofluid from the separator in liquid phase takes place in the mixer along with the flow at the pump outlet. The useful work of the turbine minus the required pumping work is the net power output of a single-flash geothermal system.

**Figure 3:**
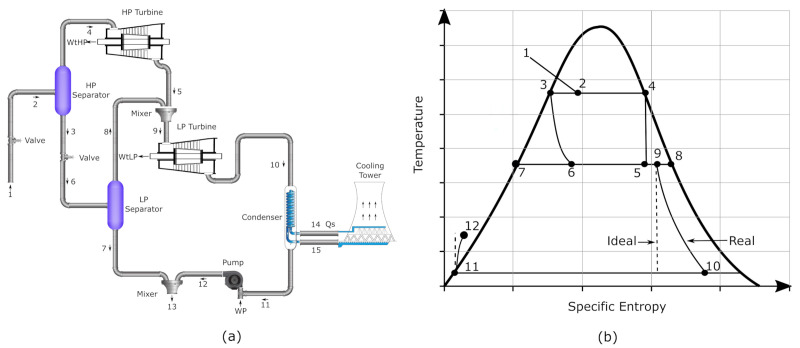
Schematic diagram of a Double-flash configuration.

Figure 3a shows a schematic diagram of a double-flash geothermal system and the entropy vs temperature scheme is presented in Figure 3b for the processes involved in the cycle. This system consists of two expansion process to generate useful work, impurity free water is used as the working fluid in the entire cycle. The main components in the double-flash system are a valve, a high-pressure separator, a low-pressure separator, a high-pressure turbine, a low-pressure turbine, a condenser, a pump and two mixers. In the valve, the geofluid is taken to high pressure in the first separator, at this point the two-phase flow is separated in liquid and vapor steam. The vapor steam at high pressure is expended, leaving the high-pressure turbine, while the liquid phase is strangled by a valve to low-pressure. Then, this two-phase flow is further separated to low-pressure in the second separator changing into vapor and liquid saturated. The second expansion is carried out taking advantage of the vapor saturated steam and the steam after the first expansion. In this condition, the working fluid enters the condenser, the liquid flowed during the condensation is pumped and mixed with the liquid phase from the low-pressure separator. The sum of the useful work of both turbines minus the required pumping work is the net power output of the double-flash geothermal system.

For both systems, a single and a double-flash geothermal system were considered in the engineering conditions described by [11].

## 3. Mass, Energy and Exergy Balance Equations

With the aim of assessing and simulating the performance from the first and second law of thermodynamic, the following assumption are listed:

- Pressure, temperatures and characteristic of geothermal fluid values in this study are thermodynamically congruent and could be proved experimentally in geothermal power plant [13],
- Steady state condition is assumed in this parametric analysis. There is thermodynamic equilibrium from the inlets to outlets in each of the components;
- The heat and frictional pressure losses in each of the components of the entire cycle are considered negligible;
- The pressure loss for vapor steam transport before the expansion process is taken as [22]:
  $$\frac{\Delta P}{P_{out}}=0.05;$$
- In all valves, the expansion processes are isenthalpic;
- The working fluid in the single and double-flash cycle at the outlet of the separator, high-pressure separator and low-pressure separator are saturated liquid and vapor;
- Operation conditions are taken from [11] studies;
- The research is based on a constant mass flow from the geo-well. Aspects related to the geothermal well such as depth, number of wells, types of rocks in the well, or pressure in the well are not considered in this study;
- Magnetic and electric terms are not considered [23];
- The terms of kinetic and potential in the energy equation are taken negligible;
- The working fluid is considered as real fluid without impurities and minerals; therefore, the chemical effect in the exergy expression are neglected [23];
- A heuristic constant temperature of 5 K was assumed between the inlet and outlet external cooling water streams in the condenser.

Following the formulation by Almahdi et al. [24] the components of the entire system can be modelled assuming they function as control volumes. A general mass rate balance is presented as:

(1) $$\sum {\dot{m}}_{in}=\sum {\dot{m}}_{out}$$

where $\dot{m}$ is the mass flow rate in $\frac{kg}{s}$.

The general energy rate balance is shown as:

(2) $${\dot{Q}}_{in}+{\dot{W}}_{in}+\sum {\dot{m}}_{in}h_{in}={\dot{Q}}_{out}+{\dot{W}}_{out}+\sum {\dot{m}}_{out}h_{out}$$

where $\dot{Q}$ is the heat rate in kW, $\dot{W}$ is the work rate in kW and *h* is the specific enthalpy in $\frac{kJ}{kg}$.

The thermo-mechanical exergy is defined as the maximum amount of work obtained when a system is brought into equilibrium from its initial state to the reference environments [23]. The flow exergy transferred by mass can be written as:

(3) $$\dot{E}x_{i}=\dot{m}\left[\left(h_{i}-h_{o}\right)-T_{o}\left(s_{i}-s_{o}\right)\right]$$

where *i* is the state of the system and ${\dot{Ex}}_{i}$ will be the exergy evaluated at that point of operation, $h_{o}$ and $s_{o}$ are the environmental dead state defined acccording to [13]. The dead state temperature, enthalpy and entropy are fixed to $T_{o}=25$ °C, $h_{o}=317.17$ $\frac{kJ}{kg}$ and $s_{o}=1.4088$ $\frac{kJ}{kgK}$.

The exergy destruction determination is defined as:

(4) $${\dot{E}}_{D,k}=\sum_{j}\left(1-\frac{T_{o}}{\bar{T}}\right)Q_{k}+{\left(\sum_{i}\dot{E}x_{i}\right)}_{inlet}-{\left(\sum_{i}\dot{E}x_{i}\right)}_{outlet}-\dot{W}$$

where *k* refers to the *k*-th component under evaluation.

Energy and exergy assessment for single and double-flash geothermal power plants were carried out. The balance equations of the pieces of equipment are shown in Table 1. For the double-flash cycle, the mass flows at points 4 and 8 (see Figure 3) are known data from [11] database. The mass flow of point 4 is equal to that of point 5, point 8 is considered saturated steam and its enthalpy can be calculated from the pressure. The enthalpy of point 5 is calculated from the isentropic efficiency of the turbine. Equation (5) shows the energy balance that is satisfied at the mixing point.

(5) $${\dot{m}}_{9}\cdot h_{9}={\dot{m}}_{5}\cdot h_{5}+{\dot{m}}_{8}\cdot h_{8}$$

For single-flash (SF) geothermal power plant, the net power output (kW) and the overall plant efficiency (dimensionless) are suggested to evaluate the performance of the entire system. The net power output is calculated as:

(6) $${\dot{W}}_{net,SF}={\dot{W}}_{TU}-{\dot{W}}_{PU}$$

where the subscript TU and PU means turbine and pump respectively. The overall plant efficiency is determined as:

(7) $$\eta_{SF}=\frac{{\dot{W}}_{net,SF}}{{\dot{q}}_{in}}\;$$

where ${\dot{W}}_{net,SF}$ presents an the amount of the net power output that is obtained and ${\dot{q}}_{in}$ is the heat supplied from the geothermal well.

For the double-flash (DF) geothermal power plant, the overall plant efficiency is computed as:

(8) $$\eta_{DF}=\frac{{\dot{W}}_{HPTU}+{\dot{W}}_{LPTU}-{\dot{W}}_{PU}}{{\dot{q}}_{in}}$$

The total exergy destruction for single ${\dot{E}}_{D,total,SF}$ and double-flash ${\dot{E}}_{D,total,DF}$ power plants analyzed in this research is determined through the expressions shown in Table 1. The heat and power balances for each component are taken according to [21].

Additionally, the second law efficiency proposed by [15] is used according to the following expression:

(9) $$\eta_{II}=\frac{{\dot{W}}_{net}}{{\dot{X}}_{geo}}$$

where ${\dot{X}}_{geo}$ is the exergy delivered by geothermal field, and in our case approximate to ${\dot{Ex}}_{2}$.

## 4. Model Validation

The operation ranges to use in this parametric analysis and the optimum point of operation based on the first law of thermodynamics are indicated in Table 2. The temperature at point 1 is a known condition, for the present work, it is assumed as the temperature before the valve that feeds energy to the thermodynamic cycles as wellhead data. According to [25] for Cerro Prieto geothermal field, the water samples from hot springs show partial equilibrium yield geothermometer temperatures in the range from 206 to 253 ${}^{\circ }$C, and the reservoir temperature calculated with geothermometers for well samples that are in full equilibrium is above 300 ${}^{\circ }$C. Measured temperatures in the exploration and production wells are between 280 and 350 ${}^{\circ }$C. According to [13] once the working fluid exits from the resource to the surface, evaporation begins because of the pressure decrease, the pressure of the working fluid should be decreased at constant enthalpy, which is called flashing process. The distribution of mass flows from the different wells of the geothermal field to the turbines of each cycle can be exemplified with the model and data from [7].

The numerical results of the simulation code were compared with the database published by [11].

**Single-Flash Geothermal Power Configuration.** In this study, the least absolute deviation $|\phi_{net,P}-\phi_{net,HM}|$ is proposed and in accordance with [26], where the variable ${\dot{W}}_{net,P}$ represent the net power output calculated in this work, and ${\dot{W}}_{net,HM}$ the net power output by [11].

The residuals method involved the calculation of the statistical parameters of central tendency and dispersion: mean (Equation (10)), deviation (Equation (11)), considering values of net power output in MW.

(10) $$\bar{\phi }=\frac{\sum_{i=1}^{n}|\phi_{net,P}-\phi_{net,HM}{|}_{i}}{n}$$

(11) $$s_{\phi }=\sqrt{\frac{\sum_{i=1}^{n}{\left(\phi_{net,P}-\phi_{net,HM}\right)}_{i}^{2}}{n-1}}$$

**Figure 4:**
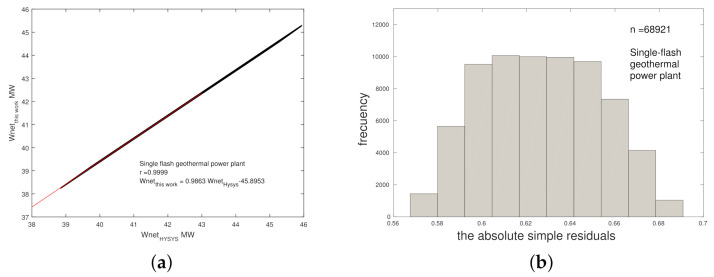
Comparison of $W_{net,HM}$ of [11] and this work for single flash geothermal power plant, (**a**) linear regression and (**b**) histogram of absolute simple residuals.

As can be seen in Figure 4a, the comparison of the net power output calculated in this work shows a similar trend with the numerical results reported by the authors. Numerical results in this work and [11] database values were compared through a linear regression model. The intercept a=−45.8953 is calculated, and the slope b=0.9863 is close to 1, therefore both indicate statistically significant correlation between the numerical results in this work and software results of [11] values without any bias. The calculated regression coefficient value is 0.9999, this value is a clear evidence of the correlation between the results. Figure 4b shows the distribution of the absolute simple error. A $\bar{\phi }$ of 0.6268 and a $s_{\phi }$ of 0.0264 were computed for n = 68,921 study cases.

Table 3 displays the numerical results of temperature, pressure, enthalpy, entropy, and exergy for the single-flash configuration of geothermal power plant assuming the optimal point described by [21]. Based on the calculations shown in the Table 3, the heat flow in the condenser, net power output of the entire system, pump power and water mass flow in the external stream of the condenser are 167,077.29 kW, 42,523.73 kW, 65.02 kW and 7992.43 $\frac{kg}{s}$, respectively.

**Figure 5:**
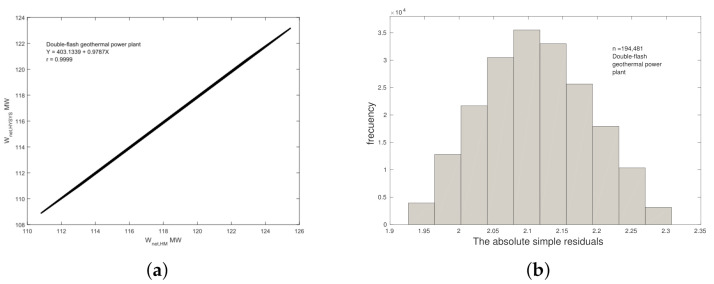
Comparison of $W_{net,HM}$ of [11] and this work for double flash geothermal power plant, (**a**) linear regression (**b**) histogram of absolute simple residuals.

**Double Flash Geothermal Power Plant.**Figure 5a shows the comparison of the net power output proposed in this model against the net power output by the Hernández-Martinez et al. Linear regression focusing on the slope and intercept values considered close to 1 and 0 respectively was carried out; as a result, the following equation was calculated: $W_{net,DF}=0.9787W_{net,HM}+403.1339$. The regression coefficient is calculated by giving a value of 0.9999. The Figure 5b shows the distribution of the absolute simple error. A $\bar{\phi }$ of 2.1111 and a $s_{\phi }$ of 0.0772 were computed for n = 194,481 study cases.

Table 4 shows the numerical results of temperature, pressure, enthalpy, entropy, and exergy for the double-flash configuration of geothermal power plant assuming the optimal point described by [21]. Based on the numerical results shown in the Table 4, the heat flow in the condenser, net power output of the entire system, pump power and water mass flow in the external stream of the condenser, high-pressure turbine power and low-pressure turbine power are 403,390.0 kW, 117,860 kW, 96.65 kW, 19,297 $\frac{kg}{s}$, 30,872 kW and 87,080 kW, respectively.

As can be seen that, for both configurations, single and double-flash, the behavior is similar, the discrepancies of the values with those obtained by Hernandez-Martinez et al. model could be assumed from the assumptions and considerations explained in Table 5. The net power output values obtained in this work agree with that reported by [25], which describes that the Cerro Prieto geothermal field production is generated by nine units, four 110 MW double-flash, four single-flash of 25 MW each, and one 30 MW single-flash.

**Figure 6:**
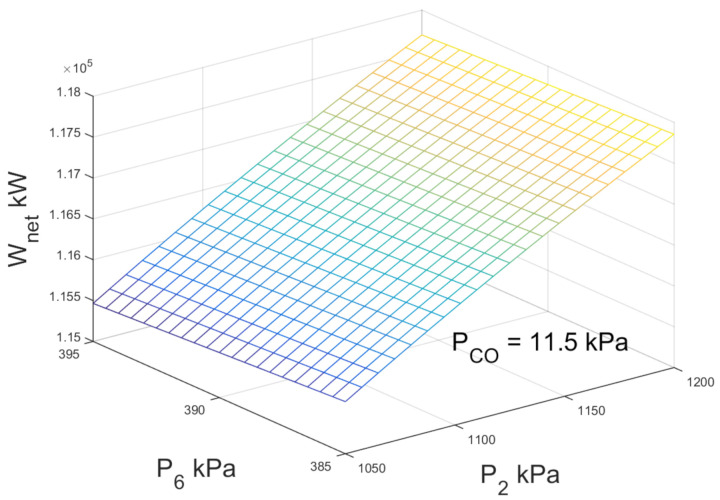
Net power output against high-pressure and low-pressure for double-flash geothermal power configuration.

According to [13], for the case of double-flash geothermal power plant, the second flashing pressure is one of the operating parameters that determines the efficiency and power production. Figure 6 shows the variations of net power output with high-pressure and low-pressure flashing. For the operation conditions described in Table 2, no optimal point is observed compatible with the research of [13], for the simulation the well temperature and condenser pressure are fixed. If the high-pressure flashing increases, the net power output increases favorably, this is due to the increase of enthalpy in the vapor steam from the high-pressure separator to the high-pressure turbine. It was observed that the net power output practically remains constant when the low-pressure flashing changes from 385 kPa to 395 kPa.

Based on the information plotted in Figure 4 and Figure 5, the model agrees with the results by [11], and it has been improved according the considerations shown in Table 5. The development shown in this work does not require commercial software that involves a license fee; this is also a contribution of this work which suggests the use of free access GNU Octave.

## 5. Results and Discussion

This section describes the following stages: (1) Parametric analysis of the exergy destruction, the effect of operating variables on the exergy destruction of both models and (2) Multiple linear regression models to calculate the total exergy destruction.

According to the information and simulation presented in Figure 6 the maximum production of electrical energy does not depend only on one variable, which brings about a problem with a set of possible solutions according to the operation parameters that were set.

The Table 6 shows the main efficiency results according to the Equations (7)–(9), and some operation parameters calculated.

As can be seen in Table 6, the calculated value for first law of efficiency is 0.1888 agrees with the results published by [11] for Cerro Prieto power plant Mexico, which it is interesting to compare this value with those reported by other authors, for example, Jalilinasrabady et al. [14] report a first law efficiency value of 7.32% and total net power produced by plant of 31,105 kW, then Bina et al. [17] report a first law efficiency of 6.22% and total net power produced by plant of 31,400 kW, both studies conducted at Salaban power plant in Iran. The results obtained by [11] are the product of simulations and estimates of the production conditions of Cerro Prieto using ANSYS software; therefore, it is necessary to thoroughly corroborate and contrast with measurements in-situ.

### 5.1. Calculation and Description of Exergy Destruction

Taking into account the operation range and optimal point operation suggested by [11], calculation and description of exergy destruction are presented aimed at understanding the entire cycles and each one of the components, as well as their sensibility to variations of operation pressure levels.

**Figure 7:**
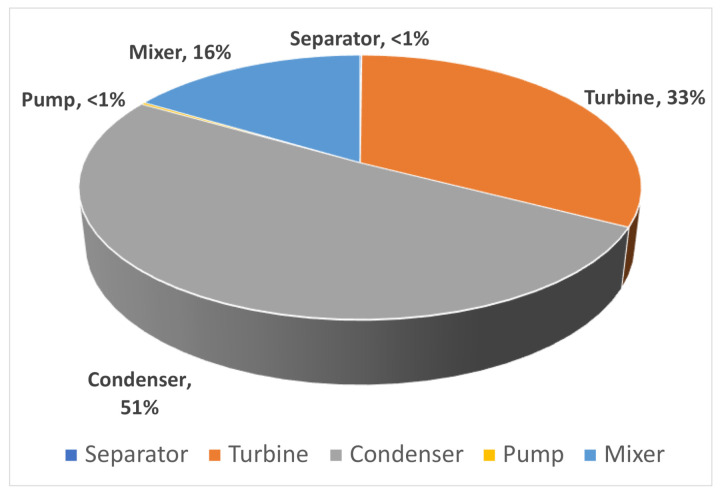
The percentage contribution components on total exergy destruction of each of the single-flash geothermal power system compounds for several configurations.

**Single-flash geothermal power plant.**Figure 7 shows the contribution in percentages for single-flash geothermal power plant in the optimal point described in Table 2. It can be seen that the percentage of contribution of the condenser was found to be up to 50.72%, followed by the turbine with a 32.54%, the mixer with a 16.36%, while the separator and pump presented a lower contribution to 0.27%. The exergy destruction for the condenser was calculated in 10,857.52 kW, for the turbine in 6965.092 kW, for the mixer in 3502.84 kW and the total exergy destruction of entire cycle in 21,404.872 kW, with the net power output of 42,523.73 kW.

**Figure 8:**
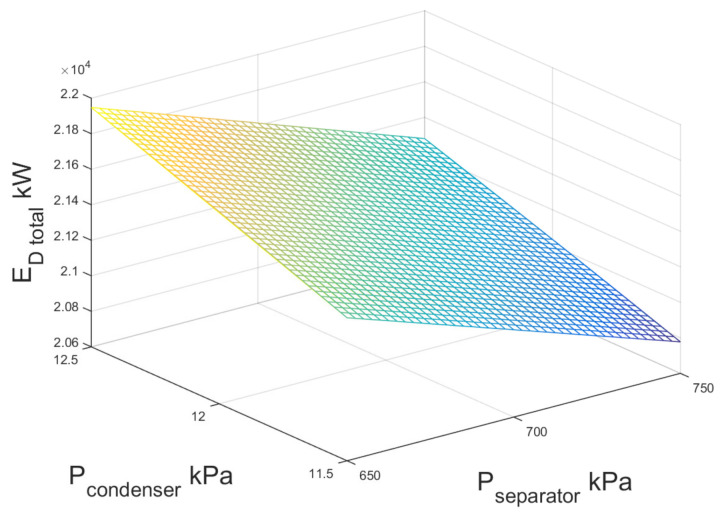
Total exergy destruction against condenser and separator pressure for single-flash geothermal power plant.

Figure 8 shows the total exergy destruction of the entire system as a function of the condenser and single flashing pressure assuming the range described previously. It was observed that when the flashing pressure decreases from 750 kPa to 650 kPa the total exergy destruction increases practically linear considering a condenser pressure to 11.5 kPa. It means that the exergy destruction is a function of the pressure level selected to separate the two-phase flow of the working fluid. The exergy destruction of the entire system decreases by 2.75% when the condenser pressure was reduced from 12.5 to 11.5 kPa. Practically, the pressure in the condenser does not influence the exergy destruction of the entire cycle. The point with the lowest total exergetic destruction of the single-acting cycle is at a condenser pressure of 11.5 kPa and a separator pressure of 750 kPa; these operating conditions are recommended in the operation of the cycle.

**Figure 9:**
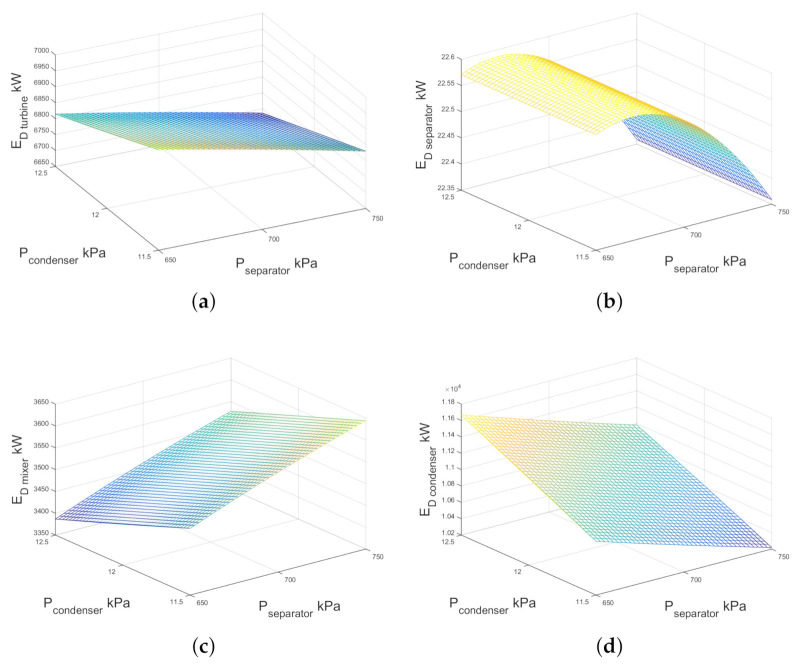
Exergy destruction against condenser and separator pressure for (**a**) turbine, (**b**) separator (**c**) mixer and (**d**) condenser for a single-flash geothermal power plant.

With respect to the single-flash geothermal power plant, Figure 9 shows the exergy destruction of each component. In Figure 9a the exergy destruction of the turbine increased when the separator pressure was decreased and consequently the quality of the two-phase flow decreased. As can be seen, the exergy destruction of the turbine slightly increases when the condenser pressure decreases, the minimum point of exergy destruction was observed at maximum condenser and separator pressures under the study operation interval. The Figure 9b displays the numerical results of exergy destruction of the separator by increasing the condenser and separator pressure. It is interesting to note that there is a maximum area of exergy destruction close to 670 kPa of separator pressure and this does not change in raising the condenser pressure from 11.5 to 12.5 kPa. In Figure 9c, note that by increasing the separator pressure, the exergy destruction of the mixer was increased, too; conversely when the condenser pressure increases from 11.5 kPa to 12.5 kPa, the exergy destruction decreases by ≈3.02%. Figure 9d shows the numerical results of the exergy destruction under the effect of the condenser and separator pressures. As can be seen, the highest values are those near condenser pressure of ≈12.5 kPa and separator pressure of ≈650 kPa.

**Figure 10:**
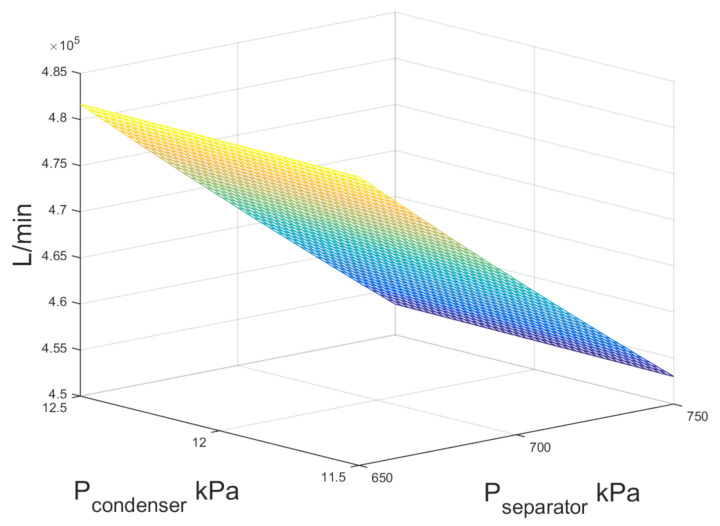
Mass flow rate of external cooling water for single-flash geothermal power plant.

The thermodynamic formulation of mass and energy balances in the condenser assuming single-flash configuration are used to calculate external water flow, it is shown in Figure 10. It is depicted in Figure 10 that the requirement of external water for the cooling of the condenser is increased by 6% as the separator pressure lowered from 750 kPa to 650 kPa due to an increase in the quality at the stream after the expansion in the turbine.

**Figure 11:**
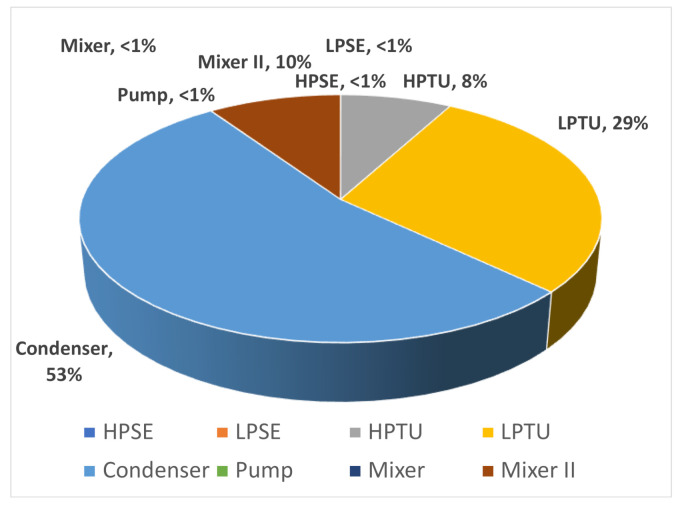
The percentage contribution components on total exergy destruction of each of the double-flash geothermal power system compounds for several configurations.

**Double-flash geothermal.**Figure 11 shows the results of exergy destruction calculated for each component of the double-flash geothermal power system, the highest contribution to the total exergy destruction is provided by the condenser, followed by the low-pressure turbine, the mixer II and the high-pressure turbine, the exergy destruction of the rest of the components is considered negligible. The total exergy destruction of the entire double-flash cycle was computed in 49.07 MW considering a net power output of 117.85 MW.

A sample condition is presented in Table 7 in which it shows the numerical results of the exergy destruction of each component of the double-flash cycle according to operating conditions in Table 4.

A parametric analysis of the total double-flash geothermal energy system and of each of the main components is carried out. The exergy destruction was calculated under the previous considerations, and keeping constant the well temperature equal to 320 ${}^{\circ }$C and the condenser pressure equal to 11.5 kPa.

**Figure 12:**
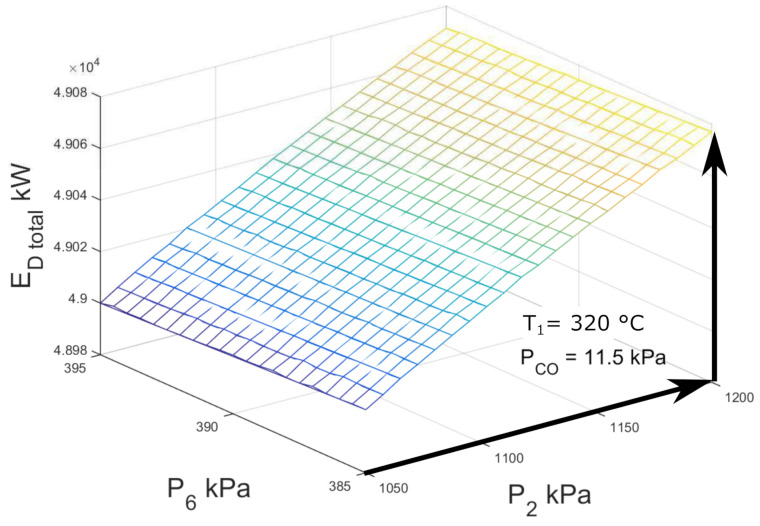
Total exergy destruction against condenser and separator pressure for double-flash geothermal power plant.

Figure 12 shows the exergy destruction of the entire double-flash geothermal power plant as indicated in the previous considerations. The sample condition is pointed out in Figure 12 based on the conditions shown in Table 7.

As may be seen, the exergy destruction of the system decreased from 49.072 MW to 49.000 MW when the high-pressure separator decreased from 1200 kPa to 1050 kPa; the exergy destruction practically remained constant when the low-pressure separator increased in the range of study. As can be seen, the exergetic destruction values are minimum when the separator pressure $\left(P_{2}\right)$ approaches 1050 kPa.

**Figure 13:**
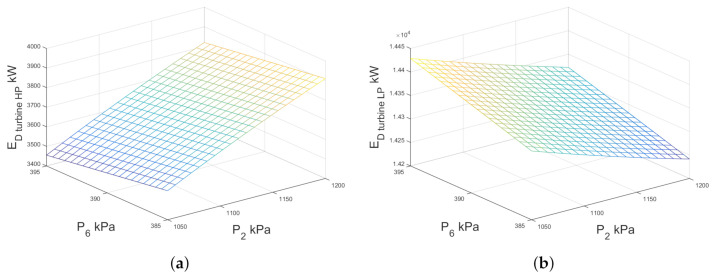
Exergy destruction against high- and low-pressure separator pressures for both turbines of double-flash geothermal power plant considering $T_{well}=320$ ${}^{\circ }$C and $P_{CO}=11.5$ kPa.

The greatest contribution of the total exergy destruction of the system is the sum of the exergy destruction of the condenser and low-pressure turbine (≈82.44%), considering the base-case illustrated in Figure 11. The Figure 13a,b show the exergy destruction of the high- and low-pressure turbines respectively. The low-pressure turbine contribution is greater than the high-pressure turbine exergy destruction. It is interesting to note that the minimum point of exergy destruction in the low pressure turbine is observed when the high pressure separator is equal to 1200 kPa and low pressure separator is equal to 385 kPa, contrary to the exergy destruction of the high pressure turbine which reaches a maximum point of 3900 kW.

**Figure 14:**
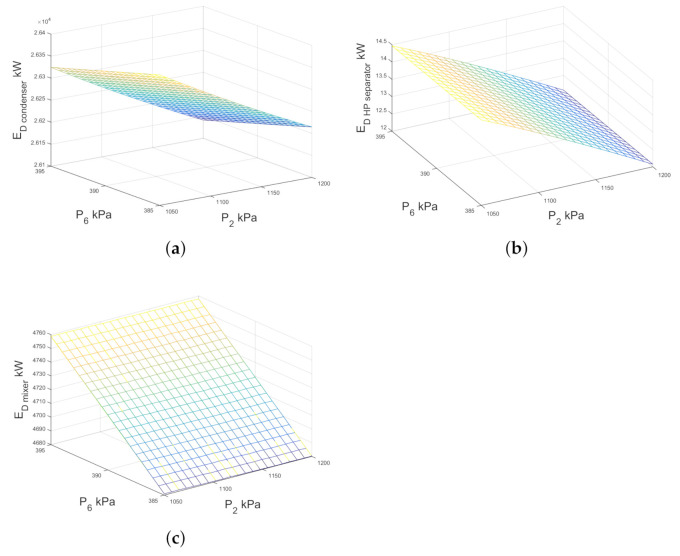
Exergy destruction against high and low pressure separator pressures for condenser, high-pressure separator and the mixer 2 of double-flash geothermal power plant.

Figure 14a–c shows the variation of exergy destruction of the condenser, the high pressure separator and the mixer 2 respectively, when the high pressure separator pressure changes from 1050 kPa to 1200 kPa and the low pressure separator pressure increases from 385 kPa to 395 kPa. Variations in pressure levels do not significantly affect the exergy destruction of the condenser, then the contribution to the total exergy destruction by the mixer is minimal. As can be seen in Figure 14b, the exergy destruction of the high-pressure separator is significantly influenced by the high-pressure separator (P2). Figure 14b shows the exergy destruction by the high pressure separator in the double-flash configuration as a function of the working pressure in both separators, the exergy destruction depends mainly on the level of pressure and it is not influenced by the pressure of the low pressure separator.

**Figure 15:**
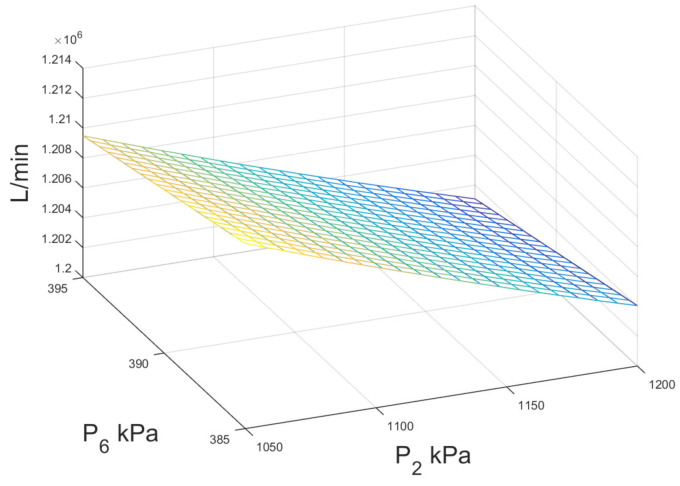
Mass flow rate of external cooling water for double-flash geothermal power plant.

With respect to the cooling water consumption in the condenser for double-flash configuration, Figure 15 shows the numerical results of external water stream when the high- and low-pressure levels were modified in the system. As can be noted, the influence of the pressure levels is not significant, when the pressure of the high pressure separator is increased from 1050 to 1200 kPa, the water consumption is increased by 0.5%, while changes in the pressure level of the low pressure separator $\left(P_{6}\right)$ practically do not affect the water consumption.

### 5.2. Multiple Linear Regression Models

According the analysis described in previous sections two database ${\dot{E}}_{total;predictedSF}$ and ${\dot{E}}_{total;predictedDF}$ were generated. In this subsection, these databases will be considered as our real models.

Now, we use a linear multiple regression method to predict the total exergy destruction (dependent variable) considering: $T_{well}$, $P_{SE}$, $P_{CO}$, and $T_{well}$, $P_{CO}$,$P_{HPSE}$, $P_{LPSE}$ as the independent variables in the single- and double-flash configurations, respectively. Table 8 displays the two new equations obtained to calculate the total exergy destruction of the single-flash ${\dot{E}}_{total;predictedSF}$ and double-flash ${\dot{E}}_{total;predictedDF}$ configurations in the geothermal power plant.

**Residual analysis.** The residual error in a LMR model is defined as:

(12) $$\varepsilon ={\dot{E}}_{total;real}-{\dot{E}}_{total;predicted}$$

The analysis of residuals allows us to validate the regression model. If the residual error in the regression model satisfies the assumptions: (a) independence, (b) normal distribution, (c) homoscedasticity (constant variance), (d) have a zero mean, (e) linear relationship and (f) little collinearity, then the model is considered valid. A graphic residual analysis method on LMR (with and without outliers) was devolved to verify possible insufficiency and violations of the assumptions. Our main sources were [27,28,29,30] and Sanford [11]. The graphic of the predicted coefficient ( $E_{total;predicted}$ ) versus standardized residuals showed that the average of residuals is zero (d), its variance is constant (c), they are independent (a) and have linear relationships (e). In contrast, the histogram of the residual errors exhibited a normal distribution with a zero mean (b), (d). To prevent the collinearity problem we used, a re-specification method was used, in an implicit manner. Therefore, it can be concluded that our linear multiple regression models, Table 8, are valid.

## 6. Concluding Remarks

The exergy analysis was successfully used to evaluate the sensitivity of the total exergy destruction to the main operation variables for a single and double-flash geothermal power plant in Cerro Prieto, Mexicali, Mexico. The following conclusions are presented. The thermodynamic model developed has been satisfactorily compared and validated with simulation data available in the literature. Further, the model proposed in this work uses the calculation of appropriate thermo-physical properties for water and steam, as well as the pressure drop for vapor transport in pipes, the above using free access software. However, it is the objective of the research group to compare the numerical results with measurements or experimental data to provide a greater degree of confidence in the prediction.

For the single-flash configuration, the total exergy destruction is a linear function of the pressure level selected to separate the two-phase flow from the geothermal field. The numerical results were performed with a base-case of $\eta_{SF}=0.1888$, ${\dot{W}}_{net,SF}=42.523$ MW, and $\eta_{II}=0.3072$. The condenser is a piece of equipment that brings more exergy destruction to the entire system ≈50.72%. For the turbine, the minimum point of exergy destruction was calculated when the condenser and separator pressure are at their highest points. According to the analysis presented, if the Cerro Prieto geothermal power plant works with high pressures to separate the geothermal fluid in steam and liquid, it determines minimum points of the exergy destruction in the condenser and separator.

For the double-flash configuration, the condenser is that heat transfer equipment that has the greatest contribution to exergy destruction ≈53.42%. The numerical results were performed with a base-case of $\eta_{DF}=0.3643$, ${\dot{W}}_{net,DF}=117.8$ MW, and $\eta_{II}=0.4983$. Additionally, based on the numerical results, it is recommended proper maintenance and the greatest investment in technology should be carried out for the low-pressure turbine, because it provides the greatest contributions to exergy destruction by about 29%. Two new equations based on multiple regression models are presented to estimate the total exergy destruction of the single and double-flash configurations in the geothermal power plant for process simulation purposes, these equations have been developed to assist design engineers in the fabrication and operation of a power plant.

Integration of aspects related to the geothermal well such as water flow, pressure, depth, types of rocks in the well, and number of wells can significantly affect the exergy destruction. Therefore, it is a line of study and research to investigate and incorporate these aspects into the model developed.

Additionally, technical aspects related to the construction and operation of the plant’s cooling towers are under investigation by the group and will be incorporated into the system model.

## Figures and Tables

**Table 1:** Exergy balance equations for the components analyzed.

Single-flash geothermal power system	
Separator $\left(SE\right)$	${\dot{E}}_{D,SE}=\dot{E}x_{2}-\dot{E}x_{4}-\dot{E}x_{3}$
Turbine $\left(TU\right)$	${\dot{E}}_{D,TU}={\dot{m}}_{4}T_{0}\left(s_{5}-s_{4}\right)$
Condenser $\left(CO\right)$	${\dot{E}}_{D,CO}=\left(\dot{E}x_{5}+\dot{E}x_{9}\right)-\left(\dot{E}x_{6}+\dot{E}x_{10}\right)$
Pump $\left(PU\right)$	${\dot{E}}_{D,PU}={\dot{m}}_{6}T_{0}\left(s_{7}-s_{6}\right)$
Mixer $\left(MI\right)$	${\dot{E}}_{D,MI}=\dot{E}x_{3}+\dot{E}x_{7}-\dot{E}x_{8}$
Total exergy destruction	${\dot{E}}_{D,total,SF}={\dot{E}}_{D,SE}+{\dot{E}}_{D,TU}+{\dot{E}}_{D,CO}+$
	${\dot{E}}_{D,PU}+{\dot{E}}_{D,MI}$
Double-flash geothermal power system	
High pressure separator $\left(HPSE\right)$	${\dot{E}}_{D,HPSE}=\dot{E}x_{2}-\dot{E}x_{4}-\dot{E}x_{3}$
High pressure Turbine $\left(HPTU\right)$	${\dot{E}}_{D,HPTU}={\dot{m}}_{4}T_{0}\left(s_{5}-s_{4}\right)$
Low pressure separator $\left(LPSE\right)$	${\dot{E}}_{D,LPSE}=\dot{E}x_{6}-\dot{E}x_{8}-\dot{E}x_{7}$
Low pressure Turbine $\left(LPTU\right)$	${\dot{E}}_{D,LPTU}={\dot{m}}_{9}T_{0}\left(s_{10}-s_{9}\right)$
Condenser	${\dot{E}}_{D,CO}=\left(\dot{E}x_{10}+\dot{E}x_{14}\right)-\left(\dot{E}x_{11}+\dot{E}x_{15}\right)$
Pump	${\dot{E}}_{D,PU}={\dot{m}}_{11}T_{o}\left(s_{12}-s_{11}\right)$
Mixer I	${\dot{E}}_{D,MI}=\dot{E}x_{5}+\dot{E}x_{8}-\dot{E}x_{9}$
Mixer II	${\dot{E}}_{D,MI}=\dot{E}x_{7}+\dot{E}x_{12}-\dot{E}x_{13}$
Total exergy destruction	${\dot{E}}_{D,total,DF}={\dot{E}}_{D,HPSE}+{\dot{E}}_{D,LPSE}+$
	${\dot{E}}_{D,HPTU}+{\dot{E}}_{D,LPTU}+{\dot{E}}_{D,CO}+$
	$+{\dot{E}}_{D,PU}+{\dot{E}}_{D,MI}+{\dot{E}}_{D,MI2}$

**Table 2:** Operation parameters for simulation.

Operation Parameter	Single-Flash	Optimal Single-Flash	Double-Flash	Optimal Double-Flash
Wellhead temperature ${}^{\circ }$C	245–255	250	315–325	320
SE pressure kPa	650–750	650	1050–1200	1200
CO pressure kPa	11.5–12.5	11.5	11.5–12.5	11.5
Low pressure SE kPa	none	none	385-395	385

**Table 3:** Thermodynamic data of the single-flash geothermal power plant assuming the optimal point conditions shown in Table 2.

State	$\dot{m}$ $\frac{\mathrm{kg}}{s}$	T ${}^{\circ }$C	P bar	*h* $\frac{\mathrm{kJ}}{\mathrm{kg}}$	*s* $\frac{\mathrm{kJ}}{\mathrm{kg}K}$	$\dot{\mathrm{Ex}}$ kW
2	420.00	161.9863	6.5000	1089.5123	2.8940	138,399.4195
3	337.97	161.9863	6.5000	684.2156	1.9628	68,224.5604
4	82.02	161.9863	6.5000	2759.5950	6.7321	70,152.2887
5	82.02	48.5682	0.1150	2240.3538	7.0168	20,598.4420
6	82.02	48.5682	0.0115	203.3511	0.6854	8355.5338
7	82.02	48.5682	6.5000	204.1439	0.6877	8363.7200
8	420.00	140.2702	6.5000	590.4633	1.7418	73,085.4348
9	7992.43	25.0000	1.0132	104.9292	0.3672	785,681.3235
10	7992.43	30.0000	1.0132	125.8337	0.4368	787,066.7022

**Table 4:** Thermodynamic data of the double-flash geothermal power plant assuming the optimal point conditions shown in Table 2.

State	$\dot{m}$ $\frac{\mathrm{kg}}{s}$	T ${}^{\circ }$C	P bar	*h* $\frac{\mathrm{kJ}}{\mathrm{kg}}$	*s* $\frac{\mathrm{kJ}}{\mathrm{kg}K}$	$\dot{\mathrm{Ex}}$ kW
2	473.81	187.9646	12.0000	1530.0738	3.8028	236,491.7703
3	299.21	187.9646	12.0000	798.4989	2.2164	71,969.9267
4	174.60	187.9646	12.0000	2783.7691	6.5217	164,509.7687
5	174.60	142.2397	3.8500	2606.9535	6.5968	129,727.0149
6	299.21	142.2397	3.8500	798.4989	2.2432	69,587.4269
7	271.26	142.2397	3.8500	598.8209	1.7625	47,799.1667
8	27.95	142.2397	3.8500	2736.3176	6.9082	21,788.2602
9	202.55	142.2397	3.8500	2624.8052	6.6398	151,512.2752
10	202.55	48.5682	0.1150	2194.8942	6.8756	50,193.9137
11	202.55	48.5790	0.1150	203.3511	0.6854	20,634.2367
12	202.55	48.6152	3.8500	203.8283	0.6857	20,712.6483
13	473.81	102.5277	3.8500	429.6939	1.3353	63,831.5530
14	19,297.08	25.0000	1.0132	104.9292	0.3672	1,896,962.5144
15	19,297.08	30.0000	1.0132	125.8337	0.4368	1,900,307.3968

**Table 5:** Comparison between considerations made by Hernández-Martínez et al. and this work.

Consideration	This Model	Hernández-Martínez
Pressure drop due to vapor steam transport in tubing.	$\frac{\Delta P}{P_{out}}=0.05$	none
Thermo-physical properties estimation	Water and steam properties according to IAPWS IF-97 for 0–1000 bar and 0–2000 ${}^{\circ }$C	Aspen Hysys software and the Property Packages of Soave-Redlich-Kwong-Twu.

**Table 6:** Efficiency values and some important values of power plant in single and double-flash configurations.

Parameter	SF	HP	DF	LP
Steam quality at separator	0.1829	0.3685		0.0934
Steam quality at turbine exit	0.8539	0.9395		0.8349
Power used by pump $\left(kW\right)$	65.02		96.6562	
Net power output produced by plant $\left(kW\right)$	42,523.73		117,855.73	
First law efficiency	0.1888		0.3643	
Second law efficiency	0.3072		0.49835	

**Table 7:** Numerical results of the exergy analysis of double-flash power plant.

Equipment	Exergy Destruction kW
HPSE	12.07486
HPTU	3910.3969
LPTU	14,241.32841
CO	26,214.794
Pump	18.24463
Mixer 2	4680.262
Total	49,077.1015

**Table 8:** Multiple linear regression model to calculate the total reversibility.

Configuration	Equation	*r*
Single-flash	${\dot{E}}_{total;predictedSF}=-4.8633\times 10^{4}+273.0194T_{well}-6.4777P_{SE}$	0.999
	$+522.6645P_{CO}$	
Double-flash	${\dot{E}}_{total;predictedDF}=-9.2876\times 10^{4}+391.9989T_{well}+$	0.999
	$+0.4395P_{HPSE}-0.8018P_{LPSE}+1418.8P_{CO}$	

## Abbreviations

The following abbreviations are used in this manuscript:

${\dot{E}}_{D}$	exergy destruction $\left[kW\right]$
$\dot{Ex}$	exergy flow rate $\left[\frac{kJ}{s}\right]$
h	enthalpy $\left[\frac{J}{kg}\right]$
$\dot{m}$	mass flow $\left[\frac{kg}{s}\right]$
P	pressure $\left[kPa\right]$
$\dot{Q}$	heat load $\left[kW\right]$
s	entropy $\left[\frac{J}{kgK}\right]$
T	temperature [${}^{\circ }$C]
$\dot{W}$	mechanical power $\left[kW\right]$
Greek letters	
η	the overall plant efficiency
$\eta_{II}$	the second law efficiency
Subscript	
in	entry
o	references state
out	exit
Acronyms	
CFE	Comisión Federal de Electricidad (in Spanish)
CO	Condenser
CP	Cerro Prieto
CPGP	Cerro Prieto Geothermal Plant
DF	Double-Flash
GIS	Geographical Information System
HPSE	High Pressure Separator
HPT	High Pressure Turbine
LPSE	Low Pressure Separator
LPT	Low Pressure Turbine
MI	mixer
NIES	National Interconnected Electrical System
SF	Single-Flash
PU	pump
SE	separator
TU	turbine
